# Surface Treatment, Chemical Characterization, and Debonding Crack Initiation Strength for Veneering Dental Ceramics on Ni-Cr Alloys

**DOI:** 10.3390/ma18163822

**Published:** 2025-08-14

**Authors:** Blanca Irma Flores-Ferreyra, María de los Angeles Moyaho-Bernal, Héctor Nahum Chavarría-Lizárraga, Jorge Castro-Ramos, Guillermo Franco-Romero, Ulises Velázquez-Enríquez, Abigailt Flores-Ledesma, Eric Reyes-Cervantes, Ana Karina Ley-García, Estela del Carmen Velasco-León, Rosendo Gerardo Carrasco-Gutiérrez

**Affiliations:** 1 Facultad de Estomatología, Benemérita Universidad Autónoma de Puebla, 31 Poniente 1304, Volcanes, Puebla de Zaragoza 72410, Mexico; bifloresf@uaemex.mx (B.I.F.-F.); guillermo.franco@correo.buap.mx (G.F.-R.); abigailt.flores@correo.buap.mx (A.F.-L.); ana.ley@correo.buap.mx (A.K.L.-G.); estela.velasco@correo.buap.mx (E.d.C.V.-L.); rosendo.carrasco@correo.buap.mx (R.G.C.-G.); 2 Facultad de Odontología, Centro de Investigación y Estudios Avanzados en Odontología, Universidad Autónoma del Estado de México, Jesús Carranza esq. Paseo Tollocan, Col. Universidad, Toluca 50130, Mexico; uvelazqueze@uaemex.mx; 3 Instituto Nacional de Astrofísica, Óptica y Electrónica (INAOE), Luis Enrique Erro #1, Sta María Tonanzintla, Puebla de Zaragoza 72840, Mexico; nchavarrialga@gmail.com (H.N.C.-L.); jcastro@inaoep.mx (J.C.-R.); 4 Dirección de Innovación y Transferencia del Conocimiento, Benemérita Universidad Autónoma de Puebla, Prol. de la 24 Sur esq. Av San Claudio, C.U., Col. San Manuel, Puebla de Zaragoza 722570, Mexico; eric.cervantes@correo.buap.mx

**Keywords:** surface treatment, metal–ceramic debonding, Ni-Cr dental alloys

## Abstract

Despite aesthetic trends, metal–ceramic restorations continue to be widely accepted due to their durability, and variations in surface preparation process can significantly influence bond strength outcomes. The purpose of this study was to determine whether there are differences in the bond strength depending on three surface treatment protocols for veneering ceramics on Ni-Cr alloys. The following surface treatments were used: (1) control (C) (no treatment), (2) airborne-particle abrasion (APA) with 50 µm Al_2_O_3_ (G1-APA), (3) APA followed by oxidation (G2-APA-O), and (4) APA-O, with a second APA (G3-APA-O-APA). Subsequently surface roughness (R_a_ and R_z_) was evaluated using profilometry, hardness was measured through Leeb’s hardness dynamic test (HLD), morphology was investigated through scanning electron microscopy (SEM), and the chemical composition of the alloy surface was evaluated using energy-dispersive spectroscopy (EDS). After surface treatments, veneering ceramic was applied, the debonding crack initiation strength (DCIS) was investigated through the three-point bending test, failure mode was classified using a stereoscopic microscope, and chemical characterization of the fractured surfaces was performed using Raman spectroscopy (RS). For DCIS, G2-APA-O demonstrated the highest value 63.97 ± 44.40 (MPa) (*p* < 0.05). The results of this study indicate that oxidation treatment has a positive effect on the bonding strength between veneering ceramic and Ni-Cr alloys.

## 1. Introduction

Despite the current trend towards aesthetic restoration materials, metal–ceramic restorations continue to be widely accepted due to their superior mechanical properties, which confer durability [1,2,3]. A wide range of noble and semi-noble alloys have been used, but their high costs have driven the development of alternative materials, including base metal Ni-Cr and Cr-Co alloys [1]. These materials enable the provision of high-quality treatments to many patients with limited financial means worldwide [4]. Clinical studies have shown that the primary failure in fixed prostheses with a substructure and ceramic, regardless of the substructure type, is “chipping” or the fracture of a small portion of the veneering ceramic in individual crowns, without significant differences [5,6]. For implant-supported fixed partial prostheses, however, metal–ceramic restoration remains the gold standard [7].

The longevity of a metal–ceramic restoration is primarily influenced by the bond between the metal and the ceramic components [8]. This bond arises from several factors, including physical forces, chemical interactions, mechanical retention, and compression due to differences in the coefficient of linear thermal expansion between these materials [9]. Specifically, wettability is crucial for proper interaction before ceramic application, as it prevents porosity and gaps [10,11,12]. The oxide layer contributes significantly to the chemical bond at metal–ceramic interfaces, forming a complex bond due to the different phases in the oxidized region [13]. The oxide layer needs to have an optimal thickness; a layer that is absent, too thin, or excessively thick results in weak bonding [14]. Mechanical retention is achieved through surface roughness, which is generated by sandblasting with Al_2_O_3_ particles. This process creates micro-retentions, allowing the material to flow into small depressions and thereby enhancing the wettability [15]. Ideally, the coefficients of linear thermal expansion for both materials should be as similar as possible. Alternatively, the metal’s coefficient should be slightly higher than that of the ceramic. This difference allows the ceramic to remain under compressive strength at room temperature [9,16]. Studies have aimed to understand and explain the mechanisms of bonding between metals and ceramics. Some focus on alloy composition [17], while others examine manufacturing techniques [17,18,19,20,21] and surface treatments [4,22,23].

There is currently a wide variety of predominant base metal alloys available, with component percentages that vary considerably. Additionally, variations in surface preparation and the manufacturing process can significantly influence bond strength outcomes. While Ni-Cr alloys are the most commonly used, the potential for nickel to cause allergic reactions has led to increased use of Co-Cr alloys for substructures intended for ceramic coating. It is essential to continue analyzing the metal–ceramic bonding interface and extend this analysis to as many alloys as possible. As the literature indicates, results apply only to the specific alloy studied and cannot be generalized due to the influence of manufacturing techniques and brands [4,17,18,19,20]. Consequently, there is a need to generate evidence of procedures under actual dental laboratory conditions to offer practical guidelines for manufacturing crowns that are applicable to both undergraduate students and professionals.

The purpose of this study was to compare the debonding crack initiation strength (DCIS) of three surface treatment protocols in undergraduate dental laboratory conditions: (1) airborne-particle abrasion (APA) using 50 µm Al_2_O_3_ particles, (2) APA followed by oxidation (APA-O), and (3) a combination of APA, oxidation, and another round of APA (APA-O-APA) on Ni-Cr alloys. The null hypothesis proposed that there would be no difference in DCIS values between these three surface treatment protocols for veneering ceramics on Ni-Cr alloys.

## 2. Materials and Methods

**Figure 1:**
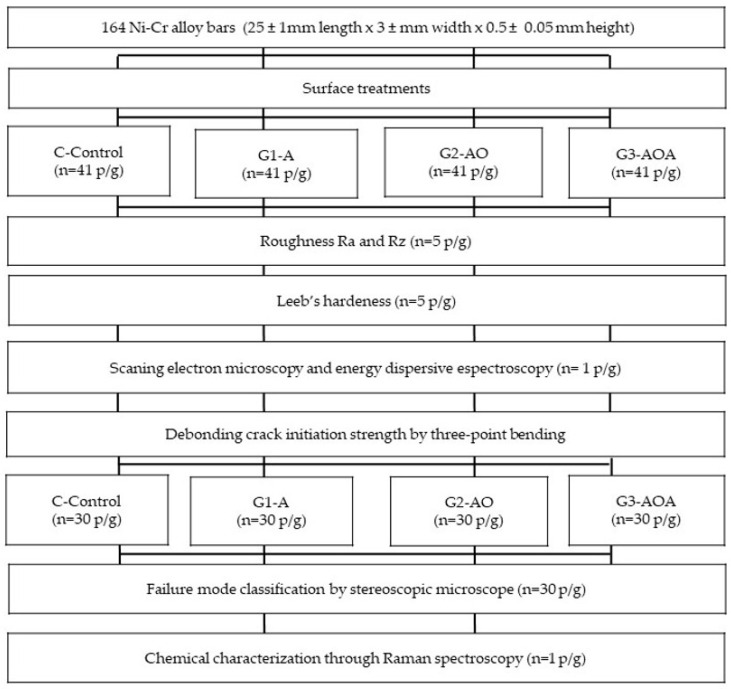
Sequence of procedure and techniques employed.

This in vitro experimental study aimed to evaluate the bond strength of the metal–ceramic interface using a Ni-Cr base alloy. The debonding/crack initiation strength test followed the procedures outlined in the international standard ISO-9693:2019(E) [24]. Figure 1 shows the study design and Table 1 provides a summary of the brand, batch number, and composition of the materials used in this study.

### 2.1. Preparation of Specimens

The Ni-Cr alloy specimens were prepared for flexural-strength testing following the ISO-9693:2019(E) [24] standards. Each specimen measured (25 ± 1) mm × (3 ± 0.1) mm × (0.5 ± 0.05) mm and adhered to the manufacturer’s instructions. The metallic material originated from the same unused batch. Castings were produced using the conventional lost wax technique. The selected dental casting alloy for metal–ceramic restorations was VeraBond^®^ (AalbaDent Inc., Fairfield, CA, USA). A template was crafted from pink calibrated wax #28 (Kemdent Associated Dental Products Ltd., Wiltshire, UK) and mounted in a silicone casting ring.

The wax templates underwent investment with a phosphate-bonded material (Smart Vest Classic, Smart Family, Aramara Dental, Jalisco, México) following the manufacturer’s guidelines. Briefly, the wax was positioned in the ring; next, 96% ethanol was applied to the wax templates and allowed to evaporate at room temperature. The investment was mixed for 60 s at a ratio of 100 g of investment to 24 mL of colloidal liquid, and it was then set at room temperature for 40 min. The wax was then calcined in a high-temperature furnace (Muffle Furnace FP31, Yamato Scientific Co. Ltd., Tokyo, Japan) at 870 °C for 60 min, with an additional 10 min for each extra cylinder using the rapid-fire investment technique.

Casting was executed using a centrifugal induction casting machine (FORNAX T, BEGO, Bremen, Germany). A preheated zircon crucible was employed, with a high fusion temperature set to 1475 °C and a 10 s heat soak. The casting was bench-cooled at room temperature without additional heat treatment. After recovering the cast, the surface underwent APA with 100 µm Al_2_O_3_ particles, and the sprues were removed using carbide cut-off discs at low speed. For finishing, all metal surfaces were ground with white aluminum oxide wheels (Mizzy Heatless Wheels, Keystone Industries, Gibbstown, NJ, USA).

After the Ni-Cr alloy specimens were completed (n = 164), specimens were randomly assigned to one of four groups using a simple randomization method implemented via a randomization application (Randomize App 0.2.6 beta, Haudainville, France). The allocation procedure involved numbering each specimen sequentially from 1 to 164. The application then generated a random sequence of these numbers. Participants were subsequently assigned to Group 1, Group 2, Group 3, or Group 4 in a cyclical manner based on their position in this randomized sequence (i.e., the first number in the sequence to Group 1, the second to Group 2, the third to Group 3, the fourth to Group 4, the fifth back to Group 1, and so on), until all 164 participants were allocated. The study involved a control group (C) and three experimental groups: Group 1 (G1-APA), APA with 50 µm Al_2_O_3_ particles; Group 2 (G2-APA-O), APA with 50 µm Al_2_O_3_ particles followed by oxidation; Group 3 (G3-APA-O-APA), APA, oxidation, and another round of APA, all on the Ni-Cr alloy. Each group consisted of 41 specimens. Within these groups, 30 specimens were allocated for debonding crack initiation strength (DCIS) determination, 5 were allocated for Leeb’s hardness dynamic test (HLD), 5 were allocated for surface roughness measurements (R_a_ and R_z_), and 1 was allocated for morphological examination using scanning electron microscopy (SEM) and energy-dispersive spectroscopy (EDS) for elemental analysis.

### 2.2. Surface Treatment and Ceramic Veneering

The Ni-Cr alloy specimens’ surfaces underwent APA using 50 µm white Al_2_O_3_ (Zeta Sand, Zhermack, Roma, Italy) at a 90° angle from a 10 mm distance, with an air pressure of 1 bar and a blaster tip with a diameter of 1 mm. The process was conducted with a speed of 30 s per cm (Eco Basic, Renfert, Hilzingen, Germany). The parameters of APA were determined according to the alloy’s manufacturer instructions and previously reported protocols [25,26]. Subsequently, the specimens were ultrasonically cleaned in 96% ethanol for 5 min and dried at room temperature (22 ± 2 °C).

In the G1-APA group, after undergoing APA and cleaning, no further treatment was applied before ceramic veneering. For the G2-APA-O group, following the same abrasion and cleaning process, the specimens underwent oxidation in a porcelain furnace (VITA V60 i-Line, Vita Zahnfabrik, H. Rauter GmbH & Co.KG, Bad Säckingen, Germany). They were fired at 980 °C in air, without vacuum, and with zero dwell time.

In the G3-APA-O-APA group, the procedure included APA and oxidation as in the previous groups. However, this group was also abraded a second time after firing using the same parameters to remove excess oxide as recommended by the alloy’s manufacturer. The specimens were then cleaned again in 96% ethanol for 5 min and dried at room temperature. The control group (C) received no additional treatment beyond the removal of investment, finishing with white aluminum oxide wheels, and ultrasonic cleaning with 96% ethanol for 5 min. After all protocols, the specimens were immediately ready for veneering.

After undergoing a specific surface treatment according to their group, the ceramic veneer was applied to the alloy specimen surfaces, covering the central area. The ceramic veneer was (8 ± 0.1) mm long and had a ceramic thickness of (1.1 ± 0.1) mm after firing. The ceramic layer was rectangular, extending the full 3 mm width of the substrate. Ceramco^®^ 3 (Dentsply International Prosthetics, York, PA, USA) in shade A2 was mixed with modeling liquid (DU, Dentsply International Prosthetics, York, PA, USA) on a clean glass surface. The mixture was then condensed by brushing, using the conventional porcelain condensation technique. Two layers of powdered opaque ceramic were applied and fired at 970 °C under a vacuum and with no dwell time. Subsequently, a third layer of dentin ceramic and a fourth layer of enamel ceramic were fired in a porcelain furnace (VITA V60 i-Line, Vita Zahnfabrik, H. Rauter GmbH & Co.KG, Bad Säckingen, Germany) at 930 °C with vacuum and a 1 min dwell time, following the manufacturer’s instructions. Table 2 display the firing schedules. Excess liquid was traditionally controlled, and a single operator fabricated the ceramic veneer under actual dental laboratory conditions. All ceramic materials were sourced from the same batch, and the most common shade was employed.

### 2.3. Surface Roughness

After the surface treatment, five samples were randomly selected from each experimental and control group (pg/n = 5). The surface roughness parameters R_a_ (the average distance from the profile to the mean line over the evaluated length) and R_z_ (the measurement of the absolute heights of the five highest profile crests and of the depths of the five lowest profile valleys within the assessed length) were measured according to ISO-4287:1998(E) [27] using a roughness tester (SJ-301, Mitutoyo, Yokohama, Japan). The measurements utilized a cut-off of 0.8 mm with a silica tip with a diameter of 2.5 µm, applying a Gaussian filter. Six independent measurements were collected from each sample, resulting in a total of 30 measurements per group.

### 2.4. Leeb’s Hardness Dynamic Test

The hardness for each group (pg/n = 5) was determined using Leeb’s dynamic test method with a portable hardness tester (TIME TH-140, Time Group, Beijing, China), following the ASTM A956-962323 standards for metallic materials [28]. This method measures the ratio of the rebound velocity to the impact velocity of a moving impactor. Measurements were conducted under ambient laboratory conditions (22 ± 2 °C) using a spherical indenter device made of tungsten carbide, with a maximum workpiece hardness of 996 HV. The HLD parameters were as follows: kinetic impact energy of 11.5 mJ, impact velocity of 2.05 m/s, rebound velocity ranging from 0.615 to 1.8245 m/s, a maximum distance of 2.00 mm between the indenter ball and the test surface, an impactor mass of 5.45 g, and a spherical radius of 1.5 mm. Hardness was assessed at six separate points on the specimen’s surface, and Leeb’s rebound hardness was determined from the average data. The Leeb’s hardness value (HLD) was calculated internally by the instrument software using the following formula: the ratio of the rebound velocity (*Vr*) to the impact velocity (*Vi*), multiplied by a factor of 1000.

(1) $$HL=\frac{Vr}{Vi}\times 1000$$

where (*HL*) is Leeb’s hardness, *Vr* is the rebound velocity radio, and *Vi* is the impact velocity.

### 2.5. Evaluation Using Scanning Electron Microscopy and Energy-Dispersive Spectrometry

For the morphological examination, representative samples from each group, along with the control sample (pg/n = 1), were observed using SEM (JSL, JSM-6610, JEOL, Peabody, MA, USA). The examination was conducted at a 20 kV acceleration voltage and a magnification of 500×, while employing Secondary Electron Imaging (SEI). To determine the elemental composition of the surface specimens, an EDS system (Oxford INCA, Oxford Instruments, Abingdon, UK) was utilized. For each sample, three spectra were captured at 20 kV, with a working distance of 10 mm and a spot size of 60, after which the average values were calculated.

### 2.6. Evaluation of the Debonding/Cack-Initiation Strength

The initial step involved determining the fracture force following ISO 9693:2019(E) [24]. Fired specimens were placed in a universal testing machine (Instron 4465, Norwood, MA, US), with the veneering ceramic symmetrically positioned opposite the applied load. The force was applied at a constant crosshead speed of 1.5 ± 0.5 mm/min and recorded until failure occurred. The fracture force, F*_fail_* (in newtons), for specimens failing due to a debonding crack at one end of the ceramic layer, was noted. Specimens that failed by cracking in the middle of the ceramic layer were replaced. The failure criterion was established as an abrupt 10% decrease in load, based on a previous pilot test. To calculate the DCIS, the fracture force F*_fail_* was multiplied by the coefficient *k*. The coefficient *k* is a function dependent on the thickness of the metal or ceramic substrate *d_z_* (0.5 ± 0.05 mm) and the Young’s modulus *E_z_* of the metal or ceramic substrate.

The debonding/crack initiation strength τ_b_ was calculated using the following formula:

*τ_b_* = *k* × F_fail_ (2)

where F is failure (fracture force) in N (Newtons), and *k* is a function dependent on the thickness of the substrate, in this case *d_z_* 2 (0.5 ± 0.05 mm):

*k* (2) = 1695 × 10^−5^ *E_z_*^2^ − 1521 × 10^−2^·*E_z_* + 6131 (3)

where *E_z_* is the substrate’s Young’s modulus. Finally, the calculation of the bonding strength was as follows:

*τ_b_* = F*_fail_* (A · d_z_^2^ + B · *d_z_* + C) (4)

where A, B, and C, are coefficients for approximating the dependence of *k* on *d_z_* for *E_z_*. Output data: *τ_b_* (MPa).

### 2.7. Evaluation of the Failure Mode

After testing the specimens for DCIS, a single observer analyzed the fractured surfaces using digital stereoscopic microscopy at 45× (VE-S5C, VELAB, Pharr, TX, USA) to determine the failure mode. Failure modes were classified as adhesive, cohesive, or mixed, employing the modified Adhesive Remnant Index (ARI) criteria [29] to quantify the ceramic material remaining in each case. The scoring was defined as follows: Score 0 indicates that no ceramic is left on the Ni-Cr alloy bond surface (adhesive failure); Score 1 indicates that less than half of the ceramic remains on the Ni-Cr alloy bond surface; Score 2 indicates more than half of the ceramic remains (mixed failure); Score 3 indicates that all ceramic remains on the Ni-Cr alloy bond surface (cohesive failure). To ascertain whether the failure was adhesive, cohesive, or mixed, both the alloy surface and the fractured ceramic surface were examined microscopically. If even a minimal amount of oxide remained on the ceramic surface, the failure was classified as mixed.

The debonded surface of a representative sample from each group was also examined using SEM (JSM 6060LV, JEOL, Peabody, MA, USA). Additionally, a cross-sectional SEM image of the same representative samples was taken. The lateral sagittal surface was polished in grinding and polishing machine (Metpol-2V, MetLab, Corp., Niagara Falls, NY, USA) and coated with gold (Desk V, Denton Vacuum, Moorestown, NJ, USA) at 25 sputter set point, time of 60, and pressure of 193 mTorr. The examination was conducted at a 20 kV acceleration voltage and a magnification of 13× and 300× for debonded areas surfaces and 300× for cross-sectional view while employing Secondary Electron Imaging (SEI).

### 2.8. Chemical Interface Characterization After Failure Using Raman Spectroscopy

After the DCIS test, one representative sample per group was analyzed using Raman spectroscopy for chemical characterization on the fractured surface. Raman spectroscopic analysis utilized an QE65000 spectrometer (Ocean Optics, Dunedin, FL, USA) with a 785 nm excitation laser operating at 500 mW, focused at 7 mm from the sample. Twenty replicate measurements were taken across three distinct sample locations, each with a one second exposure time, and all resulting spectra were subsequently averaged. Data acquisition was managed using the Spectra Suite software 2.0.162 program, applying two-scan averaging and a two-pixel boxcar filter during the initial processing, followed by a Whitaker–Henderson smoothing filter for advanced pre-processing to optimize the signal and preserve the spectral features [30].

### 2.9. Statistical Analysis

The data analysis utilized the SPSS statistical software package, version 22.0 (IBM, Chicago, IL, USA). We assessed normal distributions using the Shapiro–Wilk method. To analyze the roughness (R_a_ and R_z_) and hardness (HLD), we conducted a one-way analysis of variance (ANOVA) followed by Tukey’s post hoc analysis. The Kruskal–Wallis test, along with the Mann–Whitney post hoc U-test, was applied to DCIS. Additionally, we examined statistical differences in failure scores using the Chi-square test with Fisher’s exact correction. For the DCIS, the power value for the sample size of this study was calculated post hoc, using the highest and lowest mean values, their respective standard deviations, α = 0.05, and β = 0.20. On the basis of these values, the power was >0.80 (effect size d = 7.4).

## 3. Results

### 3.1. Surface Roughness and Leeb’s Hardness

The results indicated that the surface treatment significantly impacted the Ni-Cr alloy specimens. Table 3 presents the roughness values for R_a_ and R_z_ (in µm) and hardness (HLD). A one-way ANOVA with Tukey’s post hoc test revealed a significant effect of the surface treatment on both surface roughness and hardness (*p* < 0.05). The highest values for R_a_ and R_z_ were observed in G1-APA, followed by G3-APA-O-APA, with no significant difference between these two groups for either parameter. The R_a_ parameter for G2-APA-O showed significant differences from all other groups, while R_z_ showed significant differences except with the control (*p* = 0.38), maintaining low values in line with this behavior. In terms of hardness, G1-APA displayed the highest value, followed by the control, G2-APA-O, and G3-APA-O-APA, respectively. There were significant differences in hardness between G1-APA and all other groups, as well as between the control and G3-APA-O-APA.

### 3.2. Surface Morphology According to Scanning Electron Microscopy and Energy-Dispersive Spectroscopy

**Figure 2:**
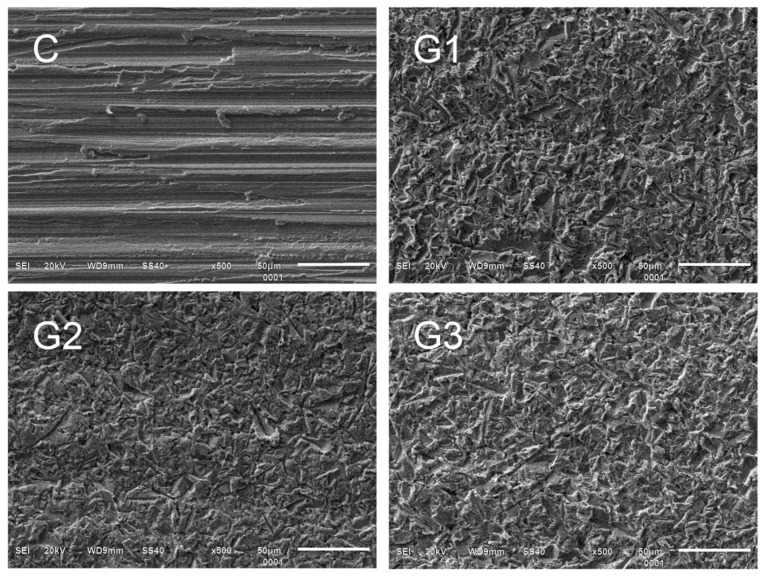
Representative SEM micrographs and secondary electron images taken at a working distance of 9 mm, spot size of 40, and magnification of 500×. For C, the surface morphology corresponds to a white aluminum oxide wheel; note the horizontal lines in one direction, and some burrs can be observed; for G1-APA, note the acute edges in a white shade; for G2-APA-O, the opaque surface can be observed, and there are no acute edges; for G3-APA-O-APA, mixed surfaces can be observed, with white acute edges and opaque obtuse edges. C: control; G1-APA: airborne-particle abrasion; G2-APA-O: airborne-particle abrasion/oxidation; G3-APA-O-APA: airborne-particle abrasion/oxidation/airborne-particle abrasion.

**Figure 3:**
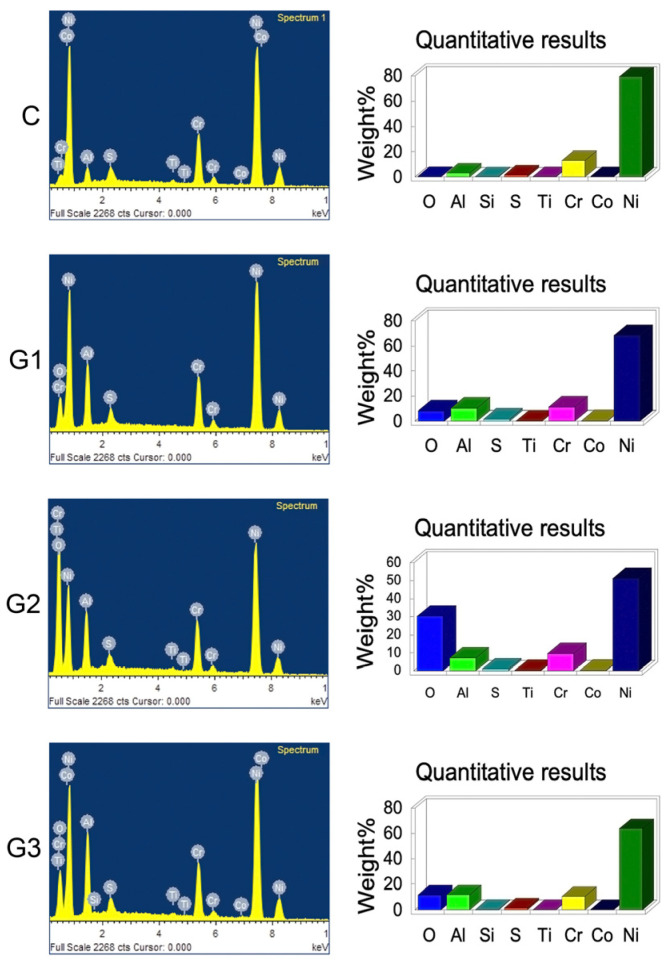
Representative EDS-evaluated sample results. The spectrum was obtained at 20 KeV with a 10 mm work distance and 60 spot sizes. The left column shows the spectrum of elements found, and the right column shows the quantitative analysis reported in weight % per group. Aluminum and oxygen increased in experimental groups, while S, Ti, Cr, Co, and Ni maintained their weight % values in all groups. The highest weight % value for O was found in G2-APA-O, and the highest weight % value for Al was found in G3-APA-O-APA. C: control; G1-APA: airborne-particle abrasion; G2-APA-O: airborne-particle abrasion/oxidation; G3-APA-O-APA: airborne-particle abrasion/oxidation/airborne-particle abrasion.

The surface morphology images (SEI) are displayed in Figure 2. A noticeable contrast exists between the control and experimental groups, though differences among the experimental groups themselves are less pronounced. Despite similarities in the micrographs, some subtle distinctions are evident. The morphology for G1-APA displays mainly acute edges, whereas G2-APA-O features obtuse edges, and G3-APA-O-APA exhibits a combination of both acute and obtuse edges. Figure 3 presents the elemental analysis of the specimen surface, detailing the weight (%) and spectrum data. The elements sulfur (S), titanium (Ti), chrome (Cr), cobalt (Co), and nickel (Ni) remain stable across all groups. In contrast, aluminum (Al) and oxygen (O) show variations in weight percentage depending on the surface treatments. The highest aluminum weight percentage is observed in G3-APA-O-APA, followed by G1-APA. For oxygen, the groups exhibit the following order of highest to lowest weight percentage: G2-APA-O, G3-APA-O-APA, G1-APA, and finally the control group (C). The elements detected align with expectations based on the alloy composition, as shown in Table 4.

### 3.3. Debonding/Crack Initiation Strength

Table 5 presents the DCIS values and the differences between groups. The highest mean DCIS value (in MPa) was observed for G2-APA-O, followed in descending order by G3-APA-O-APA, Group C, and G1-APA. The Kruskal–Wallis non-parametric test indicated a significant effect of surface treatments on DCIS between Ni-Cr alloys and the veneered ceramic (*p* < 0.05). Furthermore, the Mann–Whitney U-test revealed significant differences between groups, with G2-APA-O differing significantly from all other groups.

### 3.4. Failure Mode

**Figure 4:**
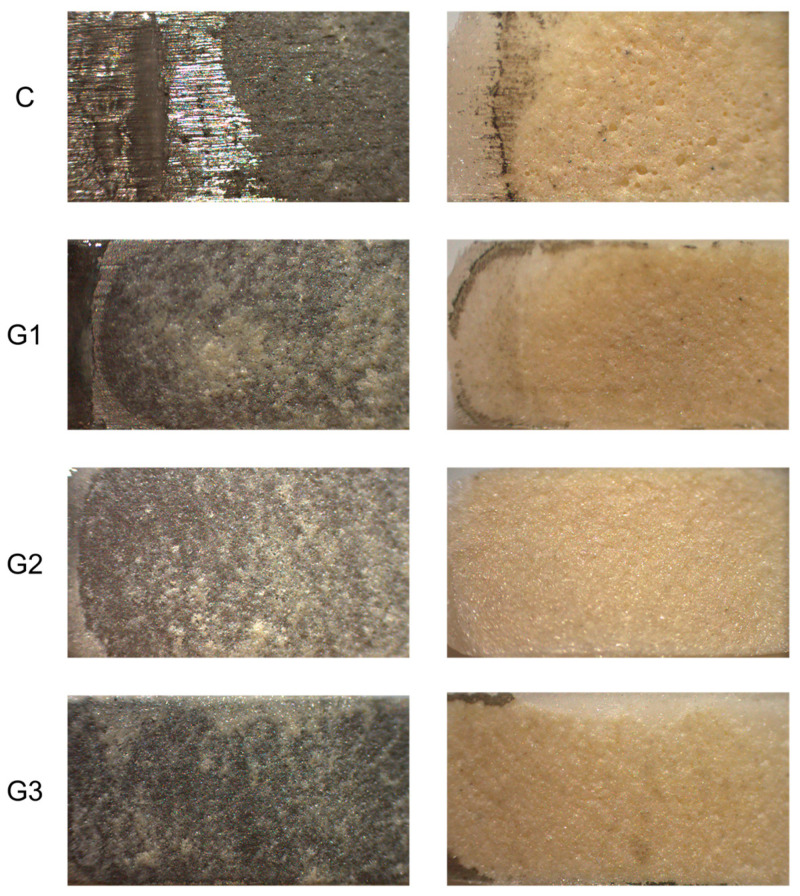
Representative stereoscopic micrographics (45× magnification) for the ARI score and failure mode evaluation of each experimental group. The letters on the left side represent the group sample, the images in the left column frame the alloy surface, and the images in the right column show a debonded ceramic surface; the ceramic and alloy surfaces correspond to each other. For C, a mixed failure could be seen; note that the Ni-Cr alloy surface is partially covered by ceramic. For the ceramic surface, oxide remnants can be seen. The G1-APA micrograph shows mixed failure, oxide lines, and stains along the surface. G2-APA-O shows cohesive failure; note that the ceramic surface is free of metallic oxides. G3-APA-O-APA also shows a mixed failure; all micrographs have an ARI score of 2. C: control; G1-APA: airborne-particle abrasion; G2-APA-O: airborne-particle abrasion/oxidation; G3-APA-O-APA: airborne-particle abrasion/oxidation/airborne-particle abrasion.

**Figure 5:**
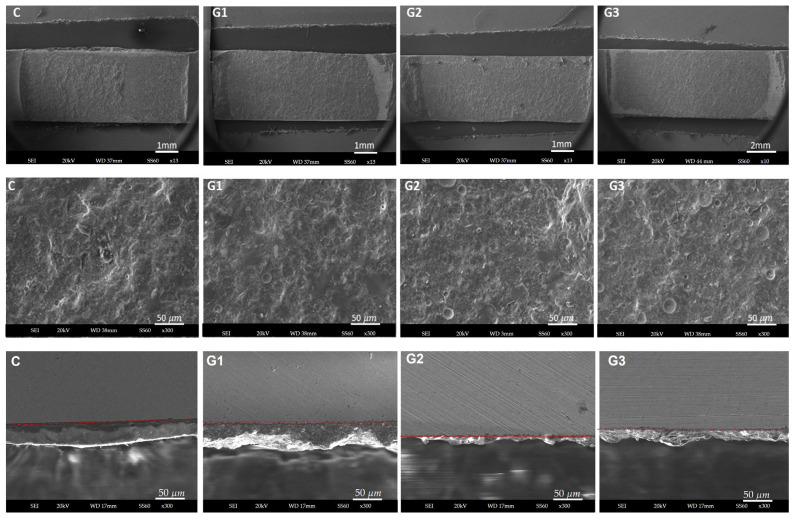
SEM images of the metal–ceramic failure surface after the debonding test. The upper images show the Ni-Cr alloy bars with the remaining veneered ceramic after failure at 13× magnification; the images in the middle row present 300× magnification micrographs of one spot in the surface area of the specimen; The images in the lower row present 300× magnification micrographs of the cross-sectional view, and the red spotted line is the boundary between alloy and ceramic interface. C: control; G1-APA: airborne-particle abrasion; G2-APA-O: airborne-particle abrasion/oxidation; G3-APA-O-APA: airborne-particle abrasion/oxidation/airborne-particle abrasion.

Table 6 shows the distribution frequency and percentages for the modified ARI scores and failure modes following the DCIS test. Mixed failure predominantly occurred with an ARI score of 2, indicating that more than 50% of the ceramic remained on the Ni-Cr alloy surfaces. This suggests that cohesive failure was more extensive than adhesive failure in most specimens. Mixed failure was noted even when only small spots of oxide were present on the ceramic surface remnant Figure 4. Cohesive failure with an ARI score of 3 appeared solely in G2-APA-O. The Chi-square comparison for the ARI scores demonstrated significant differences among the groups (Chi-square = 40.99, *p* < 0.05). Notably, there was no adhesive failure in any of the groups; mixed failure was the predominant mode, as noted in Table 7. Representative specimens after fracture were analyzed with SEM and are shown in Figure 5, where ceramic remnants can be observed. The surface morphology appears to differ from that in the SEM images before veneering as shown in Figure 2.

### 3.5. Chemical Interface Characterization

**Figure 6:**
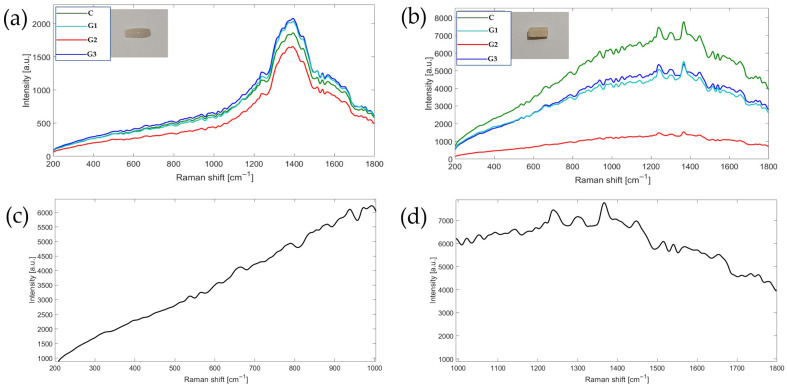
Raman spectroscopy images analysis: (**a**) image shows the spectrum of veneering enamel and dentin ceramic; (**b**) the spectrum of the debonded ceramic interface can be seen, which primarily consists of opacifier ceramic; (**c**) the spectrum of the debonded ceramic interface in the 200 to 1000 cm^−1^ region, which is the main spectral region for metallic oxides; (**d**) the spectrum of the debonded ceramic interface in the 1000 to 1800 cm^−1^ region is displayed, it corresponds to the primary spectral range for silicates. C: control; G1-APA: airborne-particle abrasion; G2-APA-O: airborne-particle abrasion/oxidation; G3-APA-O-APA: airborne-particle abrasion/oxidation/airborne-particle abrasion.

**Figure 7:**
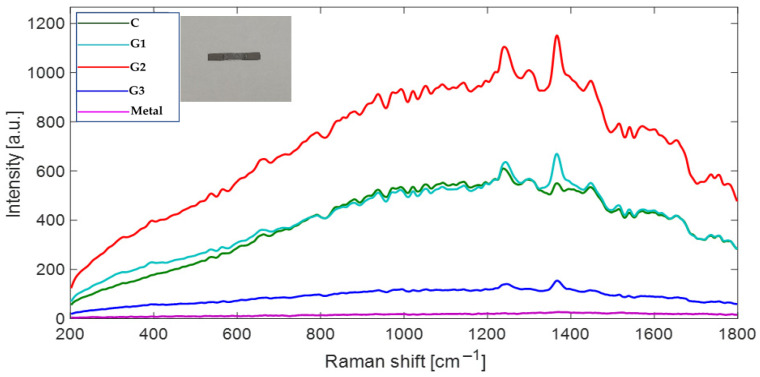
The Raman spectrum of the metal-ceramic interface. The remaining ceramic on metal alloy after debonding test was analyzed. Spectrums shows Raman bands 200–1000 cm^−1^ spectral region for metallic oxides and weak bands of Si-O, and the spectrum in the 1000 to 1800 cm^−1^ region which corresponds to the primary spectra of silicates. C: control; G1-APA: airborne-particle abrasion; G2-APA-O: airborne-particle abrasion/oxidation; G3-APA-O-APA: airborne-particle abrasion/oxidation/airborne-particle abrasion.

After conducting DCIS testing on the specimens, a representative sample was selected from each group. These samples were manually separated from the surface of the metal alloy. Once separated, Raman spectroscopy analysis was performed to determine their composition and the presence of molecular compounds. The results of the analysis are illustrated in Figure 6 and Figure 7.

In Figure 6a, the spectroscopy of the ceramic portion corresponding to the outermost part of the specimen is displayed, specifically the side opposite the adhesion area. These results show that all four specimens contained the same material, with slight variations in intensity. However, their morphology demonstrated a high correlation coefficient of 0.99.

Upon examining the composition, the spectral bands range from 420 to 740 cm−^1^, which are characteristic of Si-O-Si silica glass [31,32]. Bands between 850 and 1200 cm−^1^ correspond to Si-O stretching [33]. Additionally, there are broad bands within the spectral range of 100 to 1100 cm−^1^ that are found in dental porcelains and ceramic, and the characteristic peak of α-quartz SiO_2_ is observed at 460 cm^−1^ [34].

Figure 6b displays the spectroscopy of the ceramic surface at the metal–ceramic side interface (opaquer). It shows a gradual increase in intensity from 200 cm^−1^, peaking around 1350–1400 cm^−1^. Several defined peaks are observed between 1200 and 1500 cm^−1^, with the most prominent peak located near 1350 cm^−1^, along with multiple smaller peaks scattered throughout the spectrum, particularly between 400 and 1000 cm^−1^. Among the different groups analyzed, the G2-AO spectrum demonstrates lowest intensity compared to the others. Figure 6c shows the ceramic interface highlighting the main spectral region of the opaque ceramic. In this region, the vibrational modes characteristic of metal oxides are typically found within the range of 100 and 1000 cm^−1^, and metal–oxygen (M-O) vibrational modes usually appear between 200 and 800 cm^−1^ [35] Furthermore, Figure 6d illustrates complex stretching and strain vibrations, which can extend up to 1000–1500 cm^−1^, where silicate vibrations may overlap [34].

The Raman spectroscopy results of the remaining ceramic on the surface of the Ni-Cr alloy bar are shown in Figure 7. The presence of various metal oxides is evident. Zirconium oxide (ZrO_2_) displays Raman bands at 180, 250, 320, 370, 430, 470, and 615 cm^−1^ corresponding to its monoclinic or tetragonal phases. Titanium oxide (TiO_2_) exhibits Raman bands associated with the anatase phase at 145, 335, 395, and 515 cm^−1^. Aluminum oxide (Al_2_O_3_) shows a weak spectrum in the range of 420 to 520 cm^−1^ lacking defined peaks. The bonds between titanium and zirconium (Ti-Zr) present strong Raman bands around 700 cm^−1^, attributed to the covalent nature of their MO bonds [35]. Silica Oxide (SiO_2_) shows weak bands below 1000 cm^−1^ at approximately 230, 410, 490, and 600 cm^−1^. Silicon has strong infrared (IR) absorptions due to the ionic character of the Si-O bond, which results in weak Raman signals in the region of 700 to 1100 cm^−1^.

Among the different groups analyzed, the G2-AO spectrum demonstrates higher intensity compared to the others, while the pure metal exhibits low Raman intensity.

## 4. Discussion

This study aimed to compare three surface treatment protocols for veneering dental ceramics on Ni-Cr alloys which are commonly used in the construction of fixed metal–ceramic restorations. Metal–ceramic veneering is an adhesive process involving the union of two materials of different natures [36]. While adhesion in dentistry typically pertains to dental structures, it also plays a significant role in laboratory processes, each with unique characteristics. Due to the diverse nature of materials involved in a laboratory environment, specific protocols must be developed for each material type. Variations in composition, structure, manipulation, conditioning, or manufacturing processes can significantly impact results, as indicated by multiple studies [4,17,20,37,38,39,40,41].

In metal–ceramic adhesion, the materials involved possess distinct atomic structures: ionic for ceramics and metallic for alloys. The bonding involves chemical, mechanical, and physical processes, with metal ceramics also requiring diffusive adhesion [42,43]. Achieving effective adhesion necessitates several prerequisites, as each material’s adhesion mechanism has unique characteristics. For metal–ceramic structures, it is essential to have a regular, wettable, and clean surface with a compatible coefficient of thermal expansion, the presence of surface oxides, and appropriate surface roughness. Furthermore, research indicates that hardness may play a crucial role in the adhesion mechanism [44].

Three different protocols were compared for the treatment of Ni-Cr alloy surfaces before veneering ceramics. The focus was on understanding the effects of surface treatments on several variables, namely, roughness, as a predictor of mechanical retention and improved wettability; hardness, as a predictor of debonding strength; high-temperature treatment, to generate surface oxides for chemical interaction; and finally, the impact of these variables on DCIS, a predictor of success in both dental laboratories and clinical practice.

This study revealed significant effects on surface roughness, with G1-APA exhibiting the highest values for R_a_ and R_z_. It is important to note that all groups were abraded under identical conditions, except for Group C, which was not subjected to APA. The main variable distinguishing G1-APA from the experimental groups G2-APA-O and G3-APA-O-APA was the oxidation procedure. The differences in the results suggest that heating may affect roughness. This effect could be partially explained by the thermal treatment in Ni-Cr alloys, which microstructural analysis has shown to increase structural interaction and grain homogeneity [45].

Hardness is recognized as both a physical and mechanical property due to its role in predicting the elastic and plastic deformation resistance and the durability of dental alloys. This attribute is determined by the interatomic interactions within the material. When a load is applied, it elicits an interatomic tensile response, and if this load surpasses the material’s flexural strength, substrate deformation occurs [46]. Leeb’s hardness test was selected primarily for its portability, digital nature, and non-destructive capabilities for metallic materials. However, the most important characteristic of this technique is the fact that HLD is suitable for evaluating the restorative material’s damping capacity (the ability of a material to absorb or suppress vibrational energy) and the energy dissipation (indicates the capacity of structures to dissipate energy through the yield mechanism) [47]. Several physical characteristics are involved in a material’s strength; therefore, measuring hardness in a dynamic way is pertinent as a potential predictor of DCIS. Further studies on damping capacity and its relation to debonding strength are suggested.

Ni-Cr alloy specimens with veneered ceramics were subjected to flexural loading, generating tensile forces at the interface until failure ensued. Notably, the specimen with the highest hardness value, designated as G1-APA, also exhibited the lowest DCIS value. This finding indicates that neither higher roughness nor higher hardness proved to be a determining variable in enhancing the debonding strength in this study.

Regarding roughness, the results contrast with those reported by other authors [37,48,49]. In their studies, groups subjected to APA demonstrated greater ceramic debonding resistance. However, two of those studies involved Cr-Co alloys [37,48], and the other [49] examined Ni-Cr alloys, but without high-temperature oxidation treatment of the samples. This difference may suggest that while roughness influences the adhesion strength of the metal–ceramic complex, it is not the sole predictor. Alloy composition and oxidation are additional factors that exhibit a multifactorial influence on adhesion strength.

The DCIS assay examines flexural strength using a three-point bending test performed with a flexural-strength testing machine, with all groups prepared under identical conditions. Three different surface protocols are compared alongside a control group finished with white stone. All experimental groups underwent APA with 50 µm white Al_2_O_3_ particles under standardized conditions. Group G2-APA-O was additionally oxidized, while Group G3-APA-O-APA was oxidized and then underwent a second round of APA post-oxidation. It has been reported that chemical interactions between veneering ceramics and metallic alloys occur due to surface oxides generated during oxidation, enhancing adhesion strength between alloys and ceramics. A thin oxide layer demonstrates higher adhesion values than a thick layer [50].

Representative specimens were examined using SEM and EDS. We observed that while there was a general similarity in surface morphology among the experimental groups, the control group displayed distinct differences. The similarity among the experimental groups can be attributed to the use of the same APA protocol for all groups. However, specific morphological differences were detected. In G1-APA, we identified some acute edges. G2-APA-O exhibited obtuse edges, likely resulting from the thermal treatment [45]. G3-APA-O-APA displayed a mixed morphology with both acute and obtuse edges, which can be explained by the second APA treatment following thermal oxidation treatment. This indicates the effect of the combined treatments. The objective of a second surface treatment is to reduce the oxide layer according to the manufacturer’s instructions. However, this also alters the surface morphology, as observed in the present study. Importantly, the morphology aligns with the surface roughness results, with G1-APA showing the highest value, followed by G3-APA-O-APA and then G2-APA-O. Additionally, some black area spots were observed, which have previously been reported as aluminum oxide [37].

For EDS analysis, the elemental spectra confirmed the presence of expected elements as per the manufacturer’s reported alloy components. The elements were quantified in weight percent, with Ni being the predominant element. Alongside Cr, S, Ti, and Co, Ni remained stable across all groups. Variations in Al and O were linked to the surface treatments, and Si was identified in Groups C and G3. The highest weight percent of O occurred in G2-APA-O, followed by G3-APA-O-APA and G1-APA. These findings suggest that the increase in Al and O on specimen surfaces results from the Al_2_O_3_ treatment across all experimental groups, particularly due to the oxidation procedure, which significantly increased the oxygen content in G2-APA-O. It is feasible to differentiate the weight percent contributed by each procedure. This demonstrates how each surface treatment increases the Al and O weight percent and modifies the chemical surface characteristics of the alloy.

Chemical interaction depends on oxide thickness, which we did not measure in this study—a limitation that we acknowledge. However, following the second APA treatment for G3-APA-O-APA, which aimed to remove excess oxide as recommended by the alloy manufacturer, we observed lower DCIS values compared with the group that did not undergo a second APA treatment.

Analysis of the samples using Raman spectroscopy in the region of ceramic adhered to the metal alloy, which had been treated with three different protocols, revealed that the group subjected to airborne-particle abrasion and oxidation (G2-AO) produced a more intense spectrum compared to the other study groups (see Figure 7). This increased intensity is indicative of improved adhesion between the opaque ceramics and the metal surface, as it correlates directly with the concentration and Raman intensity of the spectra. In other words, a higher intensity indicates greater molecular interaction.

Adhesion between metals and ceramics is influenced by multiple factors. A key consideration is that metal oxides like ZrO_2_ and TiO_2_ can react with the surface of the metal, forming ionic bridges [37,38,39,40,41,42,43,44,45,46,47,48,49,50,51]. ZrO_2_ can interact with metal oxides to create intermediate Zr-O-M layers [35,52]. TiO_2_ is particularly reactive and can diffuse to form transition layers with metals such as Co, Cr, and Ni [53,54]. In contrast Al_2_O_3_ provides mechanical strength but does not promote reactions in the interfaces [55]. While SiO_2_ acts like a matrix, it does not form effective chemical bonds with metals without proper oxidative treatment, making it difficult to promote adhesion [56]. As a result, improved adhesion is primarily attributed to the active metal oxides present in the opacifier ceramic. Thermal oxidation generates a layer of compatible metal oxides (TiO_2_, Cr_2_O_3_, and NiO) on the alloy surface that chemically react with the oxides of the opacifier ceramic (ZrO_2_ and TiO_2_). The results of this study suggest that a second sandblasting can partially remove the newly formed oxide layer, as indicated by the intensity observed in the Raman spectrum (Figure 6). This removal also impacts the DCIS values, underscoring the importance of having an adequate quantity of oxides on the metal surface.

The failure mode was evaluated as previously described using a stereoscopic microscope. The stereomicroscope provided 3D visualization, retained surface information, such as color, surface texture information, and reflectivity, and enhanced viewing, time efficiency, and ease of use. Those advantages make it a valuable tool for qualitative failure analysis of materials such as dental ceramics, and the ease of use makes it possible to measure a large number of samples [57]. SEM evaluation is complementary to stereomicroscopy; as it has high magnification and resolution, it is able to perform specific site analysis and confirm findings. However, when SEM images are necessary to find traces of remnant materials on a substrate, it is considered that the failure is not cohesive, and the bond is weak [58]. The results obtained in this research showed that mixed failure was the most frequent in all groups, which indicated that there were areas of strong adhesion and some areas of weak adhesion; however, the ARI scores showed a larger extension of cohesive than adhesive failure.

The two experimental groups subjected to the oxidation process demonstrated the highest DCIS values. Chemical characterization by EDS on alloy surfaces before applying veneering ceramic revealed that the higher weight of the oxide groups resulted in greater resistance to DCIS, with Raman spectroscopy confirming this. In the failure mode analysis of G2-APA-O, a mixed ARI score of 2 occurred in 80% of specimens, with 20% showing cohesive failure. This indicates that the adhesive strength between the alloy and ceramic surpassed that within the ceramic itself. It is noteworthy that, under this study’s conditions, all protocols exceeded the minimum DCIS requirement of 25 MPa as stipulated by ISO 9396-3:2019 [21]. Consequently, all three techniques proved efficient in this in vitro investigation. Future studies should consider incorporating thermocycling or masticatory load simulations to enhance the evidence base.

## 5. Conclusions

This study highlights the significant impact of surface treatment protocols on surface roughness and hardness, revealing important insights regarding their relationship with DCIS values. While G1-APA exhibited the highest roughness and hardness values, on the contrary it had the lowest DCIS values, indicating that increased surface roughness or hardness did not inherently lead to improved interfacial strength. The analysis also showed that the APA and oxidized groups achieved higher DCIS values; however, an additional APA treatment reduced these values, suggesting a complex interaction between treatments and their effects on mechanical properties. Moreover, the results indicated a consistent occurrence of mixed failure modes across all groups, with no adhesive failures detected across the groups, highlighting the reliability of the surface treatments applied. The findings emphasize the need for further research to optimize surface treatment processes and protocols, as well as the application of aging tests. Overall, this study contributes valuable knowledge to the field of material science, underscoring the necessity for a deeper understanding of surface characteristics and their impacts on performance in practical applications.

## Figures and Tables

**Table 1:** Specifications of the materials used in this study.

Material	Composition	Batch No.	Manufacturer
VeraBond Type 5	Ni 77.9% Cr 12.6% Mo 5% Al 2.9%, Be 1.9%, Co	210712	AalbaDent, Fairfield, CA, USA
Ceramco^®^ 3 Opaque A2	Powder porcelain, Sodium Potassium aluminosilicate 60–70% WT, Tin Oxide 0–20% WT	23003177	Dentslpy Sirona, York, PA, USA
Ceramco^®^ 3 Dentin A2	Powder porcelain, Sodium Potassium Aluminosilicate 60–70% WT, Tin Oxide 0–20% WT	23003701	Denstply Sirona, York, PA, USA
Ceramco^®^ 3 Enamel Clear	Powder porcelain, Sodium Potassium Aluminosilicate 60–70% WT, Tin Oxide 0–20% WT	23003671	Dentsply Sirona, York, PA, USA
DU	Modeling liquid, Non-hazardous ingredients Mixture	18003537	Dentsply Sirona, York, PA, USA
Smart Vest	Phosphate bonded investment	F271123	Smart Family, Guadalajara, Jalisco, Mexico
Kemdent Wax	Calibrated modeling wax #28	43662	Dental Products Ltd., Wiltshire, UK
Zeta Sand	99.80% Al_2_O_3_	U112682/A	Zhermack Technical, Roma, Italy

Information provided by the manufactures.

**Table 2:** Firing schedules used in the ceramic veneering procedure.

Material	Pre-Heating Tem. (°C)	Drying Time (min)	Heating Rate (°C/min)	Final Temp °C	Holding Time (s)	Vacuum Start/Stop
Oxidation	600	1	70	980	0	No vacuum
First opaque	650	5	70	970	0	650/970
Second opaque	650	5	70	970	0	650/970
Dentine	650	3	45	930	60	650/930
Enamel	650	3	45	930	60	650/930

**Table 3:** Roughness and hardness results.

Group	R_a_ µm	R_z_ µm	HLD
C	0.91 ± 0.26	5.09 ± 1.20	575.06 ± 81.33
G1-APA	1.15 ± 0.14 *	8.14 ± 0.65 *	624.73 ± 104.0 *
G2-APA-O	0.76 ± 0.05	5.57 ± 0.52	537.73 ± 45.12
G3-APA-O-APA	1.03 ± 0.04	7.55 ± 0.74	492.73 ± 35.11

Results are expressed as means ± standard deviations. Values presented with asterisks, are significantly different according with Tukey’s post hoc (*p* < 0.05).

**Table 4:** Quantitative results (W%) in Energy-Dispersive X-ray Spectroscopy (EDS).

Spectrum	O	Al	Si	S	Ti	Cr	Co	Ni	Total
Control	0.87	3.33	0.29	1.87	0.46	13.19	0.56	79.43	100.00
G1-APA	8.23	10.13	-	1.52	0.27	11.18	0.40	68.28	100.00
G2-APA-O	30.40	7.66	-	1.07	0.26	9.51	0.35	50.74	100.00
G2-APA-O-APA	11.80	11.76	0.27	1.33	0.39	10.49	0.38	63.57	100.00

Mean values are shown, the average was obtained from 3 measurements per group. O: Oxygen; Al: Aluminum; Si: Silica; S: Sulfur; Ti: Titanium; Cr: Chrome; Co: Cobalt; Ni: Nickel.

**Table 5:** Debonding strength values.

Group	DCIS MPa
C	37.51 ± 30.81
G1-APA	33.35 ± 37.30
G2-APA-O	63.97 ± 44.40 *
G3-APA-O-APA	41.19 ± 33.88

Results are expressed as means ± standard deviations. Asterisk indicates significantly different according to Kruskal–Wallis analysis and Mann–Whitney U test (*p* < 0.05).

**Table 6:** Failure mode classified by ARI scores.

	Mixed Failure	Mixed Failure	Cohesive Failure	
Surface Treatment	Score 1 n (%)	Score 2 n (%)	Score 3 n (%)	Total
C	5 (16.6)	25 (83.3)	0	30
G1-APA	0	30 (100)	0	30
G2-APA-O	0	24 (80)	6 (20)	30
G3-APA-O-APA	11 (36.6)	19 (63.3)	0	30
Total	16 (13.3)	98 (81.7)	6 (5)	120

Score 1 and 2: Mixed failure; 3: Cohesive failure. Score 1: Mixed failure, 50% remnant ceramic material on Ni-Cr alloy surface; 2: mixed failure, with more than 50% remnant ceramic material on Ni-Cr alloy surface; 3: cohesive failure, with 100% remnant ceramic material on Ni-Cr alloy surface. Adhesive failure was not present in any specimen. Score 0: 0% remnant ceramic material on Ni Cr alloy surface. Chi-square Fisher’s exact (*p* < 0.05).

**Table 7:** Failure mode by surface treatment group.

Surface Treatment	Adhesive	Cohesive	Mixed
C	0	0	30
G1-APA	0	0	30
G2-APA-O	0	6	24
G3-APA-O-APA	0	0	30
Total	0	6	114

The mixed failure mode was predominant, and cohesive failure was only observed in G2-APA-O, which was exposed to airborne-particle abrasion and oxidation. Adhesive failure was not found. Adhesive: Fracture occurring between the ceramic and the alloy surface, with no ceramic remnants on the alloy surface. Cohesive: Fracture within the ceramic structure, with ceramic remnants covering the alloy surface. Mixed: A combination of adhesive and cohesive failure, with some areas of the alloy showing ceramic remnants and others not.

## Data Availability

The original contributions presented in the study are included in the article, further inquiries can be directed to the corresponding author.

## Abbreviations

DCIS	Debonding Crack Initiation Strength
APA	Airborne-Particle Abrasion
HLD	Leeb’s Hardness
SEM	Scanning Electron Microscope
EDS	Energy-Dispersive Spectroscopy
Al_2_O_3_	Aluminum Oxide
MPa	Megapascal
